# Erythropoietin and the effect of oxygen during proliferation and differentiation of human neural progenitor cells

**DOI:** 10.1186/1471-2121-11-94

**Published:** 2010-12-02

**Authors:** Anne-Katrin Giese, Jana Frahm, Rayk Hübner, Jiankai Luo, Andreas Wree, Moritz J Frech, Arndt Rolfs, Stefanie Ortinau

**Affiliations:** 1 Albrecht-Kossel-Institute for Neuroregeneration (AKos), Centre for Mental Health Disease, University of Rostock, Gehlsheimer Strasse 20, 18147 Rostock, Germany; 2 Institute of Anatomy, University of Rostock, Gertrudenstrasse 9, 18055 Rostock, Germany

## Abstract

**Background:**

Hypoxia plays a critical role in various cellular mechanisms, including proliferation and differentiation of neural stem and progenitor cells. In the present study, we explored the impact of lowered oxygen on the differentiation potential of human neural progenitor cells, and the role of erythropoietin in the differentiation process.

**Results:**

In this study we demonstrate that differentiation of human fetal neural progenitor cells under hypoxic conditions results in an increased neurogenesis. In addition, expansion and proliferation under lowered oxygen conditions also increased neuronal differentiation, although proliferation rates were not altered compared to normoxic conditions. Erythropoietin partially mimicked these hypoxic effects, as shown by an increase of the metabolic activity during differentiation and protection of differentiated cells from apoptosis.

**Conclusion:**

These results provide evidence that hypoxia promotes the differentiation of human fetal neural progenitor cells, and identifies the involvement of erythropoietin during differentiation as well as different cellular mechanisms underlying the induction of differentiation mediated by lowered oxygen levels.

## Background

Studies of neural stem and progenitor cells play a very important role to understand the mechanisms of differentiation of the cells into lineage specific cells like neurons and astroglia [1]. In recent years, a high number of protocols have been established for the induction of differentiation whereat the cells are generally cultured with an environmental oxygen level of 20%. But within the brain, oxygen levels are in a much lower range, and vary depending on the brain region, from 1% to 5% oxygen [2]. Therefore within the last few years more attention has been given to micro-environmental oxygen levels for optimized culturing of specific cell types, and for studying the influences of hypoxia and its underlying cellular mechanisms on growth and differentiation of stem cells [3]. Hypoxia-driven effects on regulating of stem/progenitor cell proliferation and differentiation have been shown in a number of *in vitro *systems, such as rat mesencephalic cell cultures, where hypoxia promoted neuronal differentiation [4] and hypoxia-inducible factor 1 (HIF-1) α overexpression lead to similar results as hypoxia [3]. Contrary to these previously mentioned studies in primary mouse neural stem cells, cell death was increased even though proliferation and differentiation were improved [5]. Murine neural progenitor cells (NPCs) that were exposed to hypoxia prior to engraftment into a rat brain displayed a better survival than those without hypoxic preconditioning [6]. Studer et al. [7] reported an increased number of differentiated neuronal cells and showed trophic and proliferative effects of lowered oxygen levels on rat neural precursors. Accordingly, *in vivo*, global and focal ischemia increases the proliferation and neuronal differentiation of neural stem cells in the sub-ventricular zone [8] and in the sub-granular zone of the dentate gyrus [9,10]. HIF-1α is one of the major key factors involved in the response to hypoxia and mediates a variety of cellular responses to hypoxia [3]. In hypoxic conditions HIF-1α is stabilized and induces several cellular responses such as the activation of numerous target genes e.g. erythropoietin (EPO), glycolytic enzymes, BMP, Notch and prosurvival genes [11,12] which are described to be involved in the regulation of the neuronal progenitor production with an increased neurogenesis as a part of an intrinsic hypoxia response in mice [7,13]. In our study we were interested in the effect of hypoxia on the neuronal differentiation of human NPCs. Furthermore as EPO signaling is hypoxia-inducible, we tested whether or not EPO can mimic the effects of hypoxia under normoxic conditions. Therefore we investigated the differentiation potential of human NPCs expanded and differentiated in different oxygen concentrations and the involvement of EPO in this differentiation process. As EPO is known to mimic the effects of hypoxia [7] our main objective was to demonstrate the differential effects of EPO in normoxic conditions and to illustrate that EPO causes subtle changes, but does not completely mimic hypoxia as suggested by major publications [12,13]. Moreover, we demonstrated a complex network of reactions of human NPC towards hypoxia and propose a mechanism of action within this model.

## Results

In our study we used the human immortalized neural progenitor cell line ReNcell VM (Millipore, USA). This cell line possesses the potential to differentiate into functional neuronal cells, expressing markers like βIII-tubulin and tyrosine hydroxylase. [14,15]. Furthermore the cell line is characterised by a fast proliferation [16] and a rapid onset of differentiation upon the withdrawl of growth factors [14-16]. Taken together, this cell line provides an appropriate model to study the influence of environmental conditions and factors as hypoxia or EPO, respectively.

### Stabilization of HIF-1α and EpoR expression levels in hNPCs

**Figure 1:**
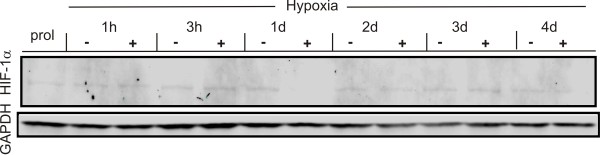
**Regulation of HIF-1α under hypoxic conditions**. HIF-1α expression at 3% O_2 _was determined after 1 h, 3 h, 1 d, 2 d, 3 d and 4 d using western blot analysis. The cells were differentiated under hypoxia up to 4 days. Application of 10 IU/ml EPO is indicated by (**+**). Untreated conditions are indicated by (**-**). GAPDH was used as loading control. "Prol" is indicating proliferating cells, representing time point zero of differentiation.

**Figure 2:**
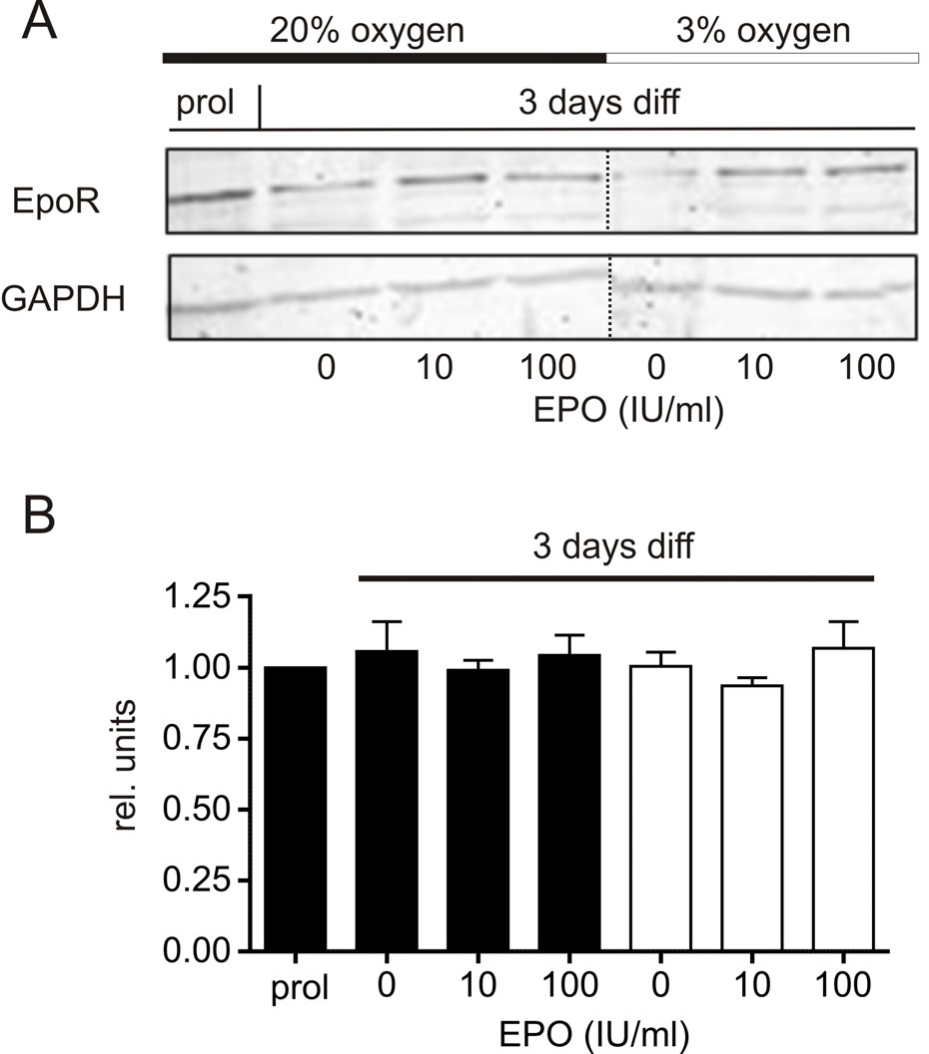
**Expression of Erythropoietin-receptor**. (A) Western blot analysis of EpoR expression levels of hNPC cell lysates at 20% O_2 _(black bars) and 3% O_2 _(white bars) in proliferating and 3 days differentiating cells. (B) Quantification of the western blot data. No significant change of the signal intensity was detected; neither between proliferating and differentiating cells nor in EPO-treated cells (mean ± SEM).

The induction of HIF-1α, a key molecule of hypoxia, is a well characterized cellular response to lowered oxygen. Therefore HIF-1α expression in hNPCs cultured at 3% oxygen over a time course of 1 h, 3 h, 1 d, 2 d, 3 d and 4 d of differentiation was measured using western blot analysis (Figure 1). EPO-treatment did not influence the expression levels of the protein. Although an early up-regulation of HIF-1α could not be quantified, the consistent expression of HIF-1α demonstrated that the system is HIF-1α sensitive. Western blot analysis of the EpoR were performed with proliferating as well as EPO-treated cells differentiated for 3 days (Figure 2A). The quantification of the data showed that the signal intensity is identical in all conditions tested, with no significant differences in the EpoR expression levels (Figure 2B), indicating that any effect of EPO would not be mediated by an upregulation of the EpoR, but by EPO itself.

### Influence of low oxygen and EPO on the proliferation rate of hNPCs

**Figure 3:**
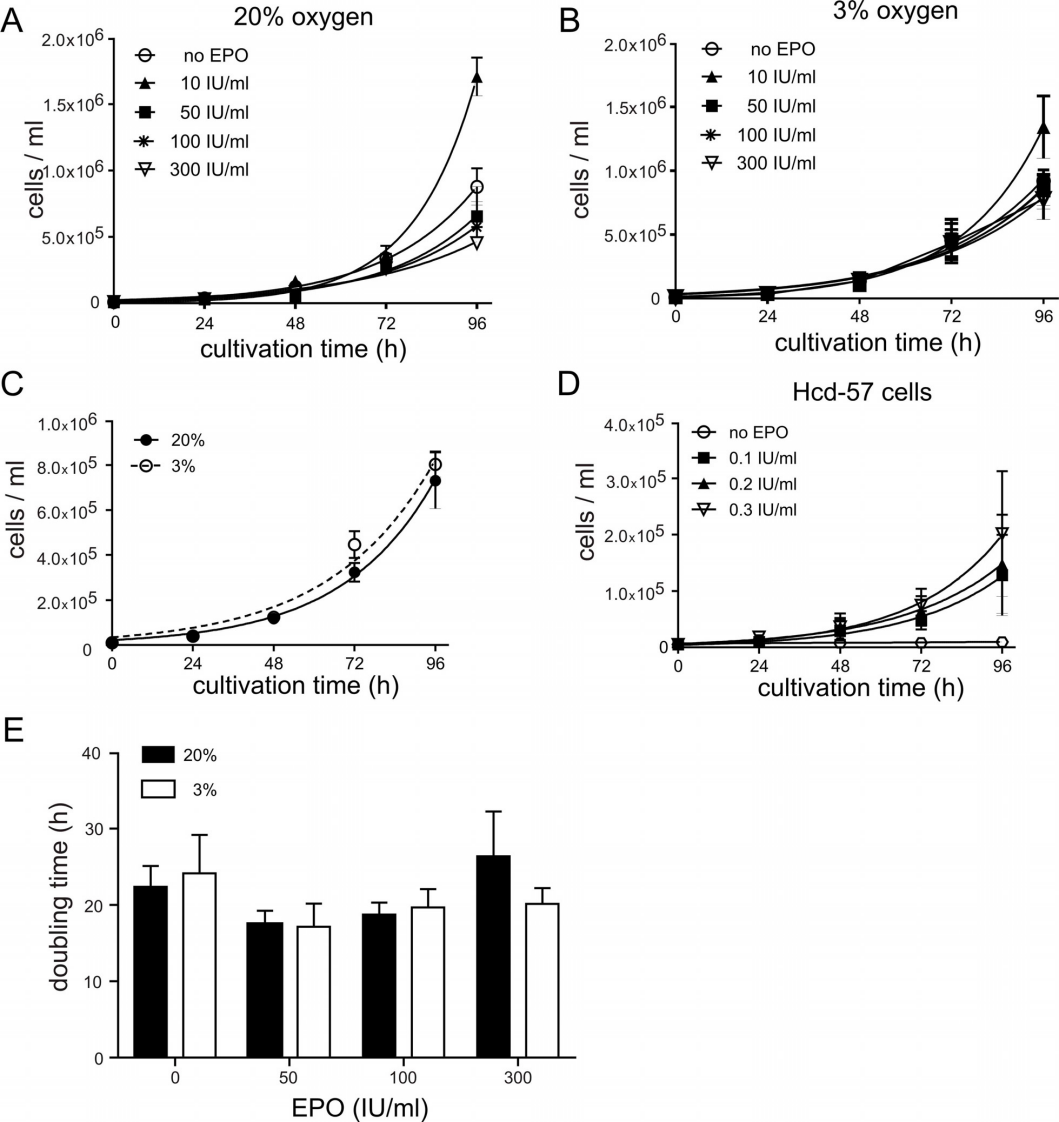
**Proliferation of hNPCs**. Effects of high and low oxygen concentration on the proliferation of hNPCs. Cells were treated with different EPO concentrations up to 4 d. Number of cells/ml was determined at 20% oxygen (A) and 3% oxygen (B). Comparing untreated cells (C), hypoxia had no impact on the proliferation rate within 4 d. The doubling times are not different between both oxygen concentrations and EPO did not influence the proliferation of cells at different concentrations (E). As control for a positive EPO-response, HCD-57 cells were used (D). Data are presented as mean ± SEM, n = 3-11 in triplicates.

To determine the effect of hypoxia on the proliferation, hNPCs were expanded either at 20% or 3% O_2_. In addition, EPO was added to proliferating cells at different concentrations and cell samples were collected every 24 h to verify the number of cells (Figure 3). At an oxygen level of 20%, EPO did not enhance cell proliferation of hNPCs compared to control cells (Figure 3A). Consistently, EPO did not change the proliferation levels of hNPCs at 3% oxygen (Figure 3B). To investigate the effect of hypoxia on the proliferation of hNPCs, untreated cells from both conditions were compared and the number of cells/ml was determined. The proliferation curves showed very similar results with no increase of the proliferation rate under hypoxic conditions (Figure 3C). The comparison of the doubling times of treated and untreated cells under normoxic and hypoxic conditions revealed no significant difference (Figure 3E). Untreated cells cultured at 20% O_2 _showed a doubling time of 19.48 ± 1.34 h and cells cultured at 3% O_2 _a doubling time of 20.45 ± 1.53 h. In addition, no significant difference of doubling times between the two groups could be detected with EPO treatment: 10 IU/ml: 11.76 ± 2.08 h (20%) versus 15.12 ± 1.94 h (3%); 50 IU/ml: 17.46 ± 1.78 h (20%) versus 19.28 ± 1.99 h (3%); 100 IU/ml: 18.77 ± 1.57 h (20%) versus 19.69 ± 4.15 h (3%); 300 IU/ml: 26.38 ± 5.86 h (20%) versus 20.57 ± 2.41 h (3%). To verify the action of EPO, HCD-57 cells, an EPO-dependent erythroleukemia cell line, were used. This cell line needs to be cultured with EPO for regular proliferation and stops proliferation when cultured without EPO. The application of EPO (0.1, 0.2, 0.3 IU/ml) resulted in a continuous proliferation of the cells, while the withdrawal of EPO stopped it (Figure 3D).

### Increased metabolic activity of NPCs by hypoxia and EPO treatment

**Figure 4:**
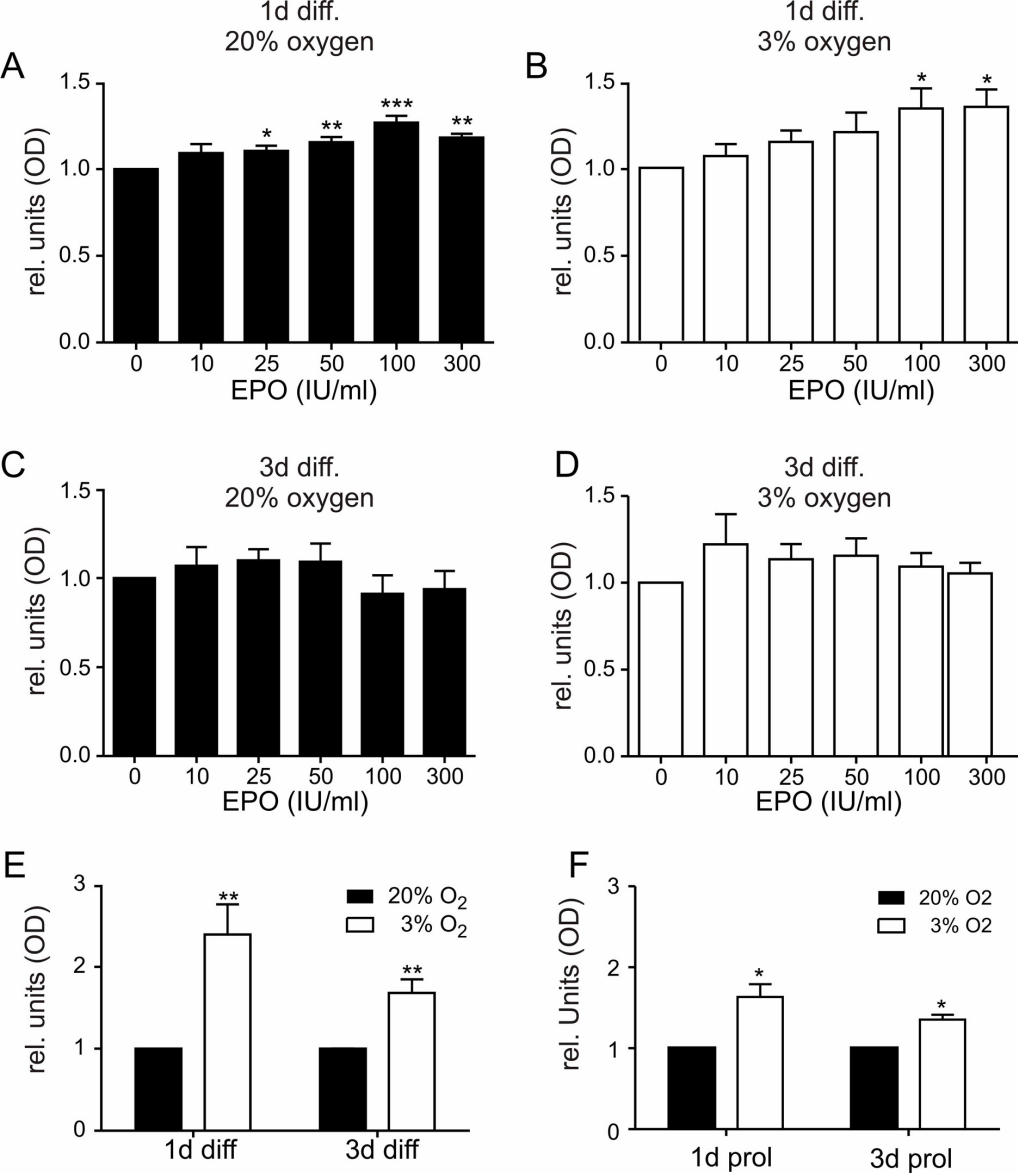
**Metabolic activity of hNPCs**. Effect of oxygen tension on the metabolic activity of differentiating hNPCs. Cells differentiated for 24 h or 72 h either at 20% (A, C, E, black bars) or 3% O_2 _(B, D, E, white bars). EPO was applied with the onset of differentiation at various concentrations (10 - 300 IU/ml). Treatment with EPO resulted in a significant increase of the absorbance from 25 IU/ml onwards at 20% O_2 _(n = 3-7) and 100 IU/ml at 3% O_2 _(n = 3-6) indicating a higher metabolic activity of cells after one day of differentiation (A, B). Samples treated with EPO and differentiated for 3 days showed similar absorbance levels at 20% (n = 3-4) and 3% oxygen (n = 3-4), whereas the increase of absorbance levels after EPO treatment were not any longer significant. Cultivation of untreated cells at low oxygen concentration resulted in a 2.4 fold increase of the absorbance after one day of differentiation and a 1.7 fold increase after 3 days of differentiation (E), indicating a higher metabolic activity (n = 4-6), compared to controls. (F) includes the metabolic activity of proliferating cells for comparison with differentiated cells (E). * p < 0.05, **p < 0.01.

In order to explore whether hypoxia or normoxia itself or in combination with EPO had effects on the metabolic activity of differentiating human NPCs, we tested the ability of mitochondrial dehydrogenases to cleave the substrate Wst-1, a tetrazolium salt derivative as a measure of metabolic activity in presence of different concentrations of EPO (Figure 4). ReNcell VM cells were incubated under differentiation conditions for 1 day and 3 days in the presence and absence of EPO, respectively. During differentiation EPO caused a significant increase of metabolic activity after 1 day under normoxic conditions from a concentration of 25 IU/ml on and higher compared to control (Figure 4A). A similar increase of the metabolic activity was observed at 3% O_2_, but higher EPO concentrations were needed for a significant change of activity (100 IU/ml and higher, Figure 4B). The significant increase of the metabolic activity caused by EPO was not any longer present after 3 d of differentiation in both conditions - normoxia and hypoxia - as seen in Figure 4C and 4D. By comparing the control values of both conditions, one can see a significant increase of the metabolic activity at 3% oxygen at both time points of differentiation, indicating a general influence of low oxygen on the cell metabolism which lasts for several days during differentiation (Figure 4E). For comparison the Wst-1 assay at 1 d and 3 d of proliferating cells is shown in Figure 4F. Consistently, hypoxia increased the metabolic activity in this condition.

### Lowered oxygen promotes neuronal differentiation of NPCs

**Figure 5:**
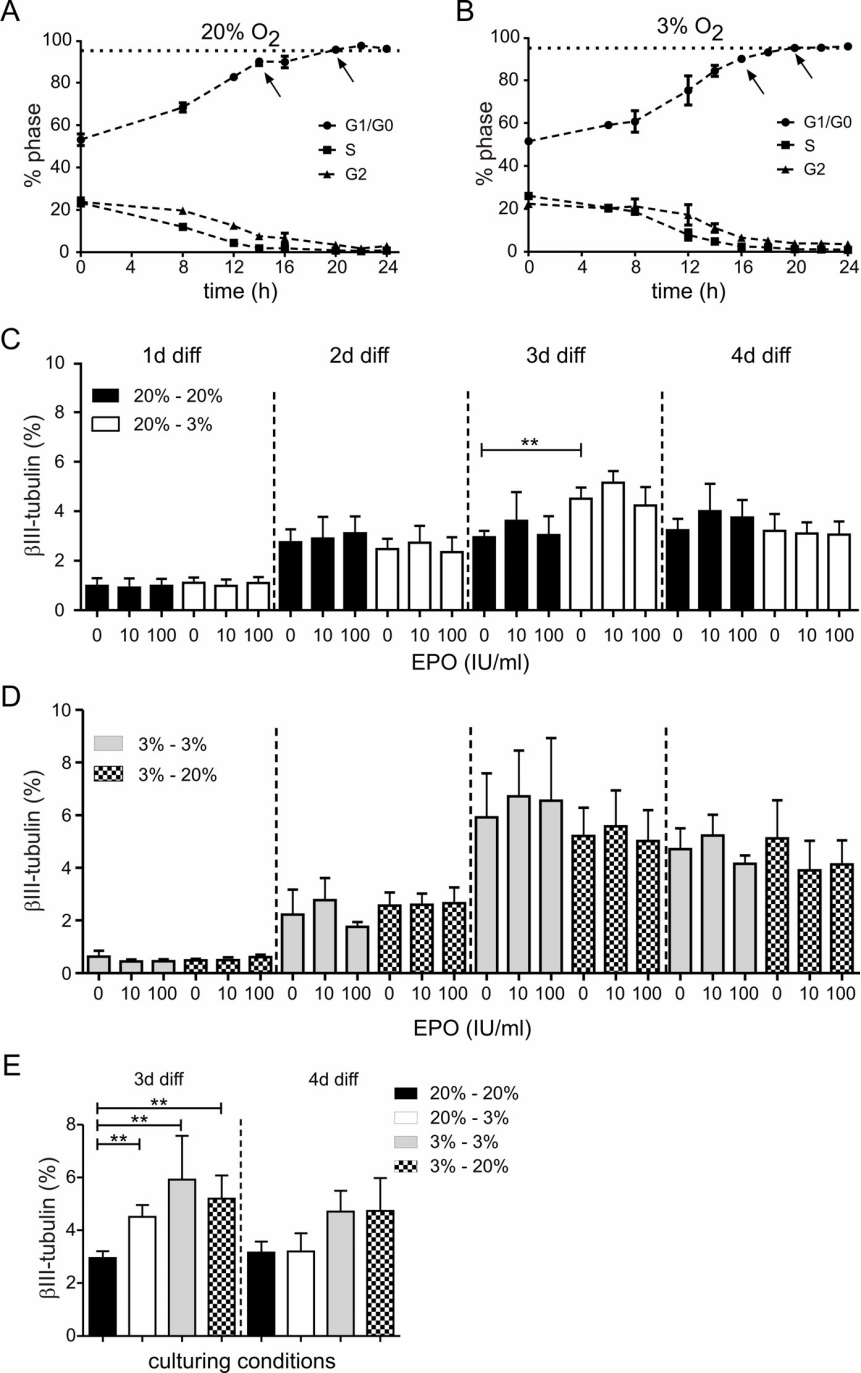
**Differentiation of hNPCs**. Differentiation of hNPCs in normoxic and hypoxic conditions. Cell cycle analysis by FACS did not reveal differences in the distribution of the proportion of cells in each cell cycle phase differentiated in 20% oxygen (A, n = 2-4 in duplicates) or 3% oxygen (B, n = 3-4 in duplicates). By measuring βIII-tub^+ ^cells up to 4 days of differentiation and EPO-treatment, the percentage of neurons was evaluated in different culture conditions. Proliferation of cells at 20% and differentiation at 20% resulted at day 3 in an increase of βIII-tub^+ ^cells compared to day one (C, n = 3-9). Cells proliferated at 3% oxygen and then differentiated at 3% and 20% oxygen showed a higher number of neurons at day 3 and day 4 (D, n = 3-5), but EPO treatment did not alter the expression levels in any of the tested conditions. (E) shows the comparison of the different control conditions, in which significant changes in the βIII-tubulin expression pattern occur with different oxygen levels (n = 3-9). Mean ± SEM, **p < 0.01). Oxygen levels during proliferation and differentiation are indicated in percentage e.g. 20% - 3% indicates 20% oxygen during proliferation and 3% oxygen during differentiation.

Next, we investigated the effect of lowered oxygen on the neuronal differentiation of human NPCs. After the withdrawal of growth factors, ReNcell VM cells were either differentiated at 20% or 3% oxygen for 4 days. First we asked the question, whether the differences of the differentiation between 20% O_2 _and 3% O_2 _is caused by changes of the proportions of cells in each cell cycle phase. Therefore we performed cell cycle measurements with flow cytometry, using the DNA binding dye propidium iodide. Figure 5 shows the percentage of cells within the phases of the cell cycle within the first 24 h of differentiation. After 20 hours, 95% of the cells reached G1/G0 phase, both in normoxic (Figure 5A) as well as in hypoxic (Figure 5B) conditions.

To verify neuronal differentiation, the expression of βIII-tubulin was measured by FACS analysis. For these experiments we included additional culturing conditions. First, the cells proliferated at 20% oxygen and were differentiated at either 20% or 3% oxygen (Figure 5C). Second, the cells were expanded at 3% and differentiated at 20% or 3% oxygen, respectively (Figure 5D). In addition, EPO was applied at 10 IU/ml and 100 IU/ml with the onset of differentiation. As shown in Figure 5C, there is no difference in the percentage of βIII-tubulin positive (βIII-tub^+^) cells between 20% and 3% oxygen and also no influence of EPO until day 3 of differentiation. At this time point, the maximal number of neurons appears with an almost twofold increase of the percentage of βIII-tub^+^cells under hypoxic conditions with 4.51 ± 0.45% compared to 2.61 ± 0.31%. At day 4 of differentiation, the level of positive cells decreases with low oxygen supply. These results indicate the supportive effect of lowered oxygen conditions for the differentiation of hNPCs. In order to determine the influence of hypoxia in detail, we cultured proliferating cells in low oxygen (3%) followed by a differentiation at 3% and 20% oxygen. In Figure 5D the percentage of neurons evaluated by βIII-tubulin expression is shown. Cells proliferated and differentiated at low oxygen levels displayed an increase of βIII-tub^+^cells at day 3 and at day 4 compared to a proliferation of cells at 20% oxygen. Next we analysed whether EPO influenced neuronal differentiation, but with both concentrations (10 IU/ml and 100 IU/ml) no change in the number of βIII-tub^+^cells was detected. Figure 5E shows a summary of 3 and 4 days differentiated hNPCs of all conditions tested. At day 3 significant differences of neuronal differentiation have been found. The number of neurons was significantly increased up to 4.51 ± 0.45% when differentiated at 3% oxygen, compared to 2.95 ± 0.25% when differentiated at 20% O_2. _In addition, the expansion of cells at low oxygen increased the number of βIII-tub^+ ^cells. When differentiated at 3%, 5.92 ± 1.66% of positive cells have been detected, when differentiated at 20%, 5.20 ± 0.87% of positive cells have been found. This indicates that there seem to be two independent mechanisms of differentiation. First, a differentiation of human progenitor cells in lowered oxygen increases the number of neurons and in addition, an expansion of cells in lowered oxygen influences the differentiation potential of hNPCs as well, independently of the culturing conditions (20% or 3% O_2_) during differentiation.

### Anti-apoptotic effect of hypoxia and EPO on differentiated hNPCs

**Figure 6:**
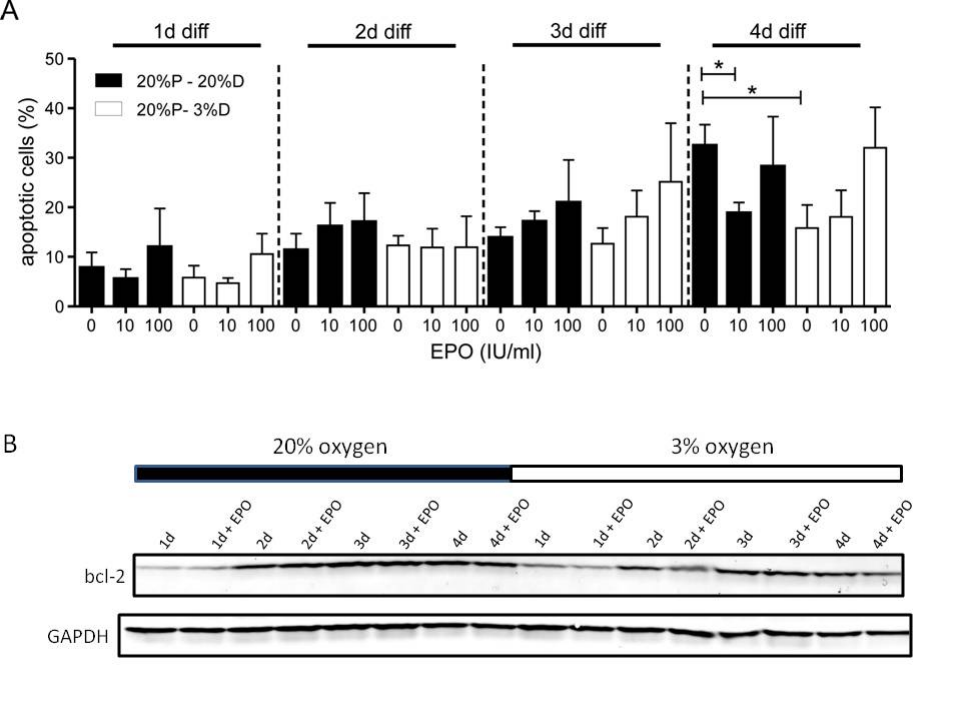
**Apoptosis in hNPCs**. Rate of apoptotic hNPCs during differentiation at 20% O_2 _(black bars) and 3% O_2 _(white bars) detected by TUNEL staining. Up to day 4 of differentiation, the percentage of apoptotic cells is continuously increasing up to 32.43 ± 4.26%, with no difference between normoxia, hypoxia, EPO-treated and control cells. At day 4 of differentiation, 10 IU/ml EPO did significantly reduce the number of apoptotic cells (18.80 ± 2.27%)%. The same decrease was observed when culturing the cells under hypoxia (15.82 ± 4.65%, A) (n = 3, mean ± SEM, *p < 0.05). Western blot analysis of Bcl-2 of hNPCs differentiated and treated with either normoxia, hypoxia or 10 IU/ml EPO over a time course of 4 days revealed an increase of signal with the onset of differentiation. GAPDH was used as loading control (B).

Since differentiation of progenitor cells is associated with apoptosis and EPO is a well known anti-apoptotic mediator, we investigated the amount of apoptotic cells during differentiation in normoxic and hypoxic conditions (Figure 6). Again the cells differentiated up to 4 days and each day samples were taken from cells cultured under normoxic and hypoxic conditions with and without the application of EPO. In order to estimate the amount of apoptotic cells in our cell population a TUNEL staining and consecutive FACS analysis was performed. Over time we observed a continuously rising apoptosis starting with 7.78 ± 3.10% that culminated in 32.43 ± 4.26% at day 4 in cells cultivated with normoxic oxygen levels. During the first three days neither hypoxia nor EPO affected the apoptosis of the hNPCs. On day 4 of differentiation we remarkably observed that both in hypoxia and normoxic EPO-treated cells the level of apoptotic cells was only half as high as in the normoxic control (hypoxia 15.82 ± 4.65%, normoxia 10 IU/ml EPO 18.80 ± 2.17%). There was no significant difference between EPO-treated normoxic cells and cells differentiated in hypoxia (p = 0.6). Application of EPO under hypoxia did not lead to an additional effect (p = 0.5). A western blot analysis was performed to measure the expression of the anti-apoptotic protein Bcl-2 in cells differentiated up to 4 days. In accordance with the finding of the FACS measurements, an increase of Bcl-2 over time was observed (Figure 6B).

## Discussion

Standard cultivation conditions for cell cultures comprise the use of 20% oxygen, nevertheless a number of studies have described an enhanced proliferation in lowered oxygen. Reducing oxygen can have a number of different effects such as the increase of proliferation as shown by Zhao et al. [3] and Studer et al. [7] for rat embryonic mesencephalic cells, or conversely a decrease of proliferation as described by Chen et al. [17] who showed that long-term proliferation in hypoxia was not beneficial for hESC with short splitting intervals. Studer et al. [7] investigated the proliferation and differentiation of embryonic mesencephalic rat cells and came to the conclusion that hypoxia was beneficial for the cells in culture and that EPO could mimic this effect under normoxic oxygen levels. Recently Santilli et al. [18] described an increased proliferation though the cell cycle remained unaffected as well as an increased neuronal differentiation and decreased cell death of human neural stem cells caused by mild hypoxia. The effects of lowered oxygen on the proliferation of stem and progenitor cells are not limited to the central nervous system [7,19]. More physiological culturing conditions are also favoured by other cell types like bone marrow stromal cells [20] and mesenchymal cells [21].

As a first step in this study we verified the expression of HIF-1α (Figure 1) and the EpoR (Figure 2). The sensibility of the hNPCs to hypoxic conditions is indicated by the expression of HIF-1α (Figure 1). A similar effect was observed by Zhou and Miller [19], Zhao et al. [3] and Zhang et al. [4] ranging from 30 minutes to 24 hours after the onset of hypoxia. HIF-1 is activated under hypoxic conditions in a variety of cell types and the HIF-1 targeted genes play an important role in maintaining cellular homeostasis in response to hypoxia [22-24]. To investigate the EpoR we chose western blotting as the currently available antibodies lead to inconclusive results obtained by immunocytochemistry [25]. The EpoR expression level was not altered by culturing the cells under EPO application or hypoxic conditions, the latter being in line with the absence of a hypoxic EPO effect. Even though this is contrary to Theus et al. [6] where hypoxia led to an increase in the EpoR expression, Milosevic et al. [26] likewise observed that hypoxia does not affect EPO signaling. This inconsistency could be due to different culturing conditions or cell types. The effect of EPO on the metabolic activity and apoptosis is independent from the regulation of expression of its receptor since the expression levels are not altered between different stages of proliferation or differentiation, as well as EPO treated cells. In summary, we conclude that the differentiation of the human NPCs used in this study as a model system is hypoxia-sensitive and EPO-responsive.

Both hypoxia and EPO have been reported to induce the proliferation of NPCs [3,5,7], though our own data support the idea that hypoxia does not change the proliferation rate or doubling times within three days of expansion (Figure 3). Similar results were obtained by Chen et al. [17] where lowered oxygen levels did not prove to be favourable, and by Milosevic et al. [27] who described a positive effect of hypoxia on the proliferation only after culturing NPCs for 1 month, but not prior to that. In addition, EPO did not affect proliferation although the EpoR could be detected in proliferating cells and 10 IU/ml EPO seems to lead to an increased proliferation though this effect was not significant compared to the control. However, higher amounts of EPO could be saturating and thus lead to no effect, either.

The differentiation of the hNPCs was investigated under various conditions. First, the metabolic activity of differentiating hNPCs was monitored with and without EPO treatment (Figure 4). An effect of EPO was detected early in 1 day differentiated cells. Remarkably at 3% oxygen, EPO was required at higher concentrations to produce an equivalent effect. This indicates that hypoxia acts only in part via the EPO pathway and that addition of EPO mimics the effect of lowered oxygen. Generally one can say that hypoxia increases the metabolic activity of hNPCs, which was highest at 1 d of differentiation, indicating the importance of early differentiation processes, as the effect at day 3 was not as high as at day 1. These data are in accordance with Studer et al. [7] where EPO mimicked the effect of hypoxia under normoxic conditions in embryonic mice NPCs.

For further investigation of the differentiation, the cell cycle of the hNPC was analysed under normoxic and hypoxic conditions (Figure 5). This analysis revealed that the cells needed around 20 h to enter G1 phase, and that this time frame is the same under normoxic and hypoxic conditions. These findings are in line with data about the cell cycle of murine midbrain NPCs where the cell cycle, the proliferation and neurosphere formation was not altered within 4 weeks of cell culture [27]. Similar results were obtained by Santilli et al. [18] who likewise demonstrated no effect of hypoxia on the cell cycle of human NSCs. These results are of major importance to further interpret the expression levels of βIII-tubulin as a marker for neuronal differentiation.

In this study EPO did not alter neuronal differentiation in the hNPCs (Figure 5). This is in contrast to rat and human mesencephalic progenitors where EPO enhanced the number of neurons [3,7]. A possible explanation for this discrepancy could be the fact that different model systems have been used. The percentage of neurons in our study was increased after culturing the cells under hypoxic conditions. This is in accordance with Zhang et al. [4] and Studer et al. [7], where hypoxic culturing conditions also led to a higher yield of neurons. Since the cells enter differentiation at the same time point under normoxia and hypoxia the higher yield of neurons is not due to an accelerated cell cycle, leading to the conclusion that hypoxia induced neuronal differentiation in the precursor cells.

An increase in neurogenesis could be obtained by two different mechanisms - one during proliferation and the other during differentiation - partially mimicked by EPO. First, culturing differentiating NPCs under lowered oxygen increased the number of neurons after 3 days of differentiation. In addition, proliferation of NPCs under hypoxia and differentiation of those cells under hypoxic or normoxic conditions raised the same amount of neurons, indicating a manipulation of the progenitor cell pool during proliferation. EPO partially mimicked the effect under normoxia and displayed anti-apoptotic effects under these culturing conditions (Figure 5E). Therefore we propose two different mechanisms of differentiation. One deals with the increase of neuronal cells by hypoxia during differentiation and the other one displays an increase of the progenitor pool of cells during proliferation under hypoxia. The two mechanisms result in the same effect, namely the increase of neuronal cells and the increase of the overall activity of differentiated cells. The first mechanism indicates that hypoxia induces differentiation and the second one indicates that hypoxia increases the pool of differentiating cells by changing the cell-fate of the progenitor cells. Proliferation was investigated at 3% O_2 _and the rate of differentiation did not change when cells were differentiated at 3% as well. These results demonstrate that 3% oxygen modifies the differentiation capability of NPCs.

The cell line used in this study showed a maximal number of neurons of around 6% (Figure 5), which can be interpreted as a limitation of this study, however reported levels of neurons in other NPC lines are similar. Nevertheless, this cell line also possesses advantages like the very fast differentiation potential and the easy accessibility, which enabled us to closely monitor changes in proliferation and differentiation. Therefore, those cells serve as a model to investigate differentiation mechanisms which then can be transferred to systems which allow for an engraftment into the CNS to cure neurodegenerative diseases like Parkinson's disease or stroke.

Concerning apoptotic cells, the number was reduced by 50% at day 4 of differentiation at 3% oxygen (Figure 6). This apoptotic effect was not in consensus with a neuronal cell death, as the number of neurons was not influenced which leads to the conclusion that the number of βIII-tub^+ ^cells at 3 days of differentiation is not only an outcome of an anti-apoptotic effect. At the fourth day of differentiation the effect of EPO is anti-apoptotic, but numbers of neuronal cells are not altered by EPO and therefore EPO has no neuron-specific anti-apoptotic effect. We observed an increased apoptosis at day 4 in the cells that underwent proliferation and differentiation at 20% oxygen, however the underlying mechanism is not clear. Depending on the severity of hypoxia it can have differential effects on the apoptosis. On the one hand it was proven to be anti-apoptotic in a model for hypoxic-preconditioning [6], on the other hand it can be pro-apoptotic if it is lowered beyond levels of mild hypoxia [5,18]. In our study, the anti-apoptotic effect of hypoxia was also indicated by the expression of the anti-apoptotic protein bcl-2. The western blot of bcl-2 revealed an increase between day one and two of differentiation, followed by a stable expression level (Figure 6). Shingo et al. [13] showed an increase of neurons induced by hypoxia. This enhancing effect was mimicked by EPO, as it promoted the production of neuronal progenitors. This is contrary to our results, as EPO could not manipulate the neuronal-producing effect of hypoxia, but did mimic other effects of hypoxia, like the anti-apoptotic effect during differentiation. The percentage of cells rescued by EPO (18.80 ± 2.27%) at 20% oxygen was not significantly different from the amount of cells rescued by hypoxia (15.82 ± 4.65%) proving that EPO has the potential to imitate hypoxic effects under normoxia.

Contrary to Studer et al. [7] and Shingo et al. [13], EPO did not completely mimick the actions of hypoxia in our study. In this study, a human fetal cell line was used whereas Studer et al.[7] and Shingo et al. [13] used mouse embryonic stem cells. This leads to the conclusion that either the point in time (fetal vs. embryonic) or the origin (human vs. mouse) can account for the observed differences. In addition, the application of human recombinant EPO to murine cells might lead to different results than in the human system. And finally, the oxygen concentration can also influence the outcome as shown by Zhang et al. [4] and Horie et al. [5]. Both tested varying oxygen concentrations ranging from 0% to 10% and found 2% to 3% oxygen to be most effective.

For translational and clinical research our findings are important because we provide further evidence of increased neurogenesis in hypoxic scenarios. The cell survival and ideal environmental oxygen after engraftment of hNSC remain yet unclear and our data supports the thesis that a hypoxic environment, as seen in stroke or other neurodegenerative diseases, are beneficial for engrafted hNSC. Furthermore we were able to provide evidence that hypoxia could induce neurogenesis during proliferation and differentiation, thus the engrafted cells would not have to be used at a certain point in time during the cell cycle and therefore making the engraftment process easier. Researchers have tried to profit from EPO as a neuroprotective agent [28] in patients with stroke [29] but it remains unclear how EPO acted neuroprotective. There are three main theories of EPO action in the human brain. The first presumes a better oxygenation of the brain through an elevation of red blood cells after EPO application, the second assumes EPO effects on astrocytes and blood vessels and indirectly affecting neurons and the third theory actually proposes a neuroprotective effect of EPO [28]. We provide supporting evidence for the last theory, which encourages the use of EPO in stroke. As EPO mainly acts in hematopoiesis and can thus cause hematopoietic side effects, the neuroprotective effect we explored for hNSCs should be further and directly exploited by derivatives of EPO, which are non-hematopoietic, neuroprotective and able to pass the blood brain barrier easily. Such structural as well as functional variants of EPO [30] that fulfil these requirements, among them modified antibody fragments [31] and peptides [32], have been described recently.

## Conclusions

**Figure 7:**
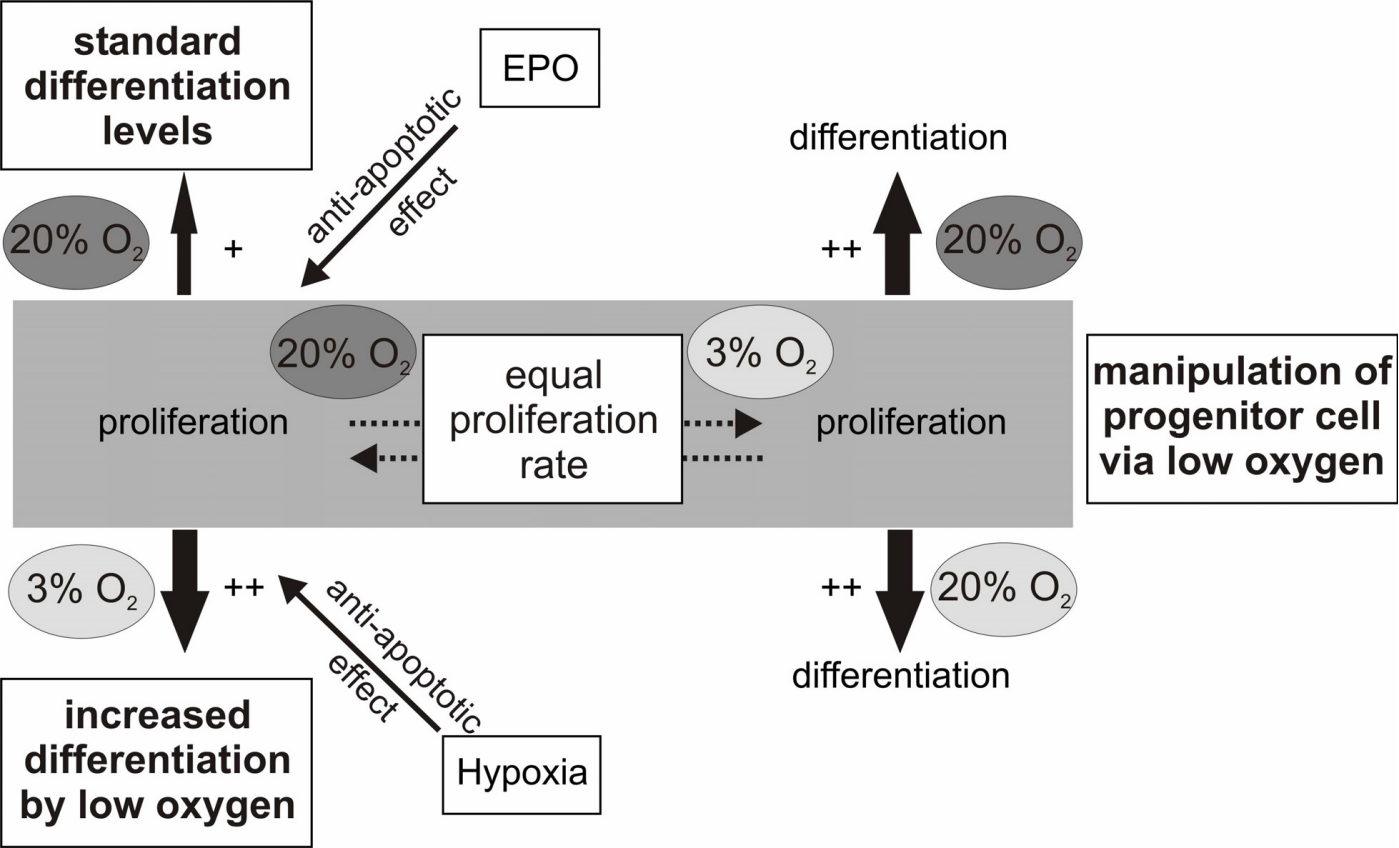
**Summary of the effects of EPO and hypoxia**. Potential mechanisms of neuronal differentiation of hNPCs and the influence of hypoxia and EPO. Proliferating cells in normoxia and hypoxia possess the same proliferation rate. Proliferation and differentiation of cells under normoxic conditions leads to an average yield of neurons. However, changing the oxygen tension after proliferation to hypoxia will lead to a higher yield of neurons. Similar results can be obtained by culturing the cells in hypoxic conditions during proliferation, though a subsequent change to normoxia or continuing the cell culture in hypoxia leads to no additional advantage for the hNPCs. We conclude that hypoxia can induce neurogenesis directly during differentiation and also influence the pool of progenitor cells during proliferation. Anti-apoptotic effects of hypoxia also play a major role in the neuroprotective properties of hypoxia. This anti-apoptotic effect as well as the increased metabolic activity can be partially mimicked by EPO.

In summary, we provide evidence for an important role of hypoxia in the differentiation of human NPCs and the modulatory action of EPO in vitro. Figure 7 outlines a hypothetical model of the action and interaction of hypoxia and EPO including the underlying cellular mechanisms. Hypoxia displays two modes of action. First, the proliferation and expansion of NPCs under hypoxic conditions increases neuronal differentiation. Second, hypoxia displays an anti-apoptotic action effecting the entire cell population thus leading to an increased survival rate after the induction of differentiation. EPO partially mimicked these hypoxic effects during differentiation and in addition, protected the differentiated cells from apoptosis. In summary, we conclude that the presented data support further research for the treatment of neurodegenerative diseases as EPO is acting anti-apoptotic in human NPCs. This also encourages the thesis that EPO can be directly used for treatment of stroke or neurodegenerative diseases as we provide evidence for a direct effect of EPO on neuronal cells.

## Methods

### Cell culture of NPCs

In this study we used the human fetal neural progenitor cell line ReNcell VM (Millipore, USA). Cell culture was carried out as described previously [15,16]. Cells were cultivated on laminin (Trevigen, Gaithersburg, MD, USA) coated dishes at 37°C in 5% CO_2 _in DMEM/F12 media supplemented with Glutamax, B27 media supplement, heparin sodium salt and gentamycine (all Invitrogen, Karlsruhe, Germany). Epidermal growth factor (20 ng/ml; EGF; Roche, Mannheim, Germany) and basic fibroblast growth factor (10 ng/ml; bFGF; Roche, Mannheim, Germany) were added to the media during proliferation. To induce differentiation, growth factors were removed from the media. For a decreased oxygen level of 3% an adjustable incubator was used and the oxygen level was lowered with N_2_. For application studies, EryPo (EPO, Janssen-Cilac, Germany) was applied once in two different concentrations (10 IU/ml and 100 IU/ml) with the induction of differentiation.

The murine EPO-dependent erythroleukemia cell line HCD-57 was used as positive control for EPO treatment [33]. These cells were grown in suspension in RPMI medium supplemented with 10% FCS and 1% gentamycine and variable concentrations of EPO.

### Cell proliferation assay

The performance of the electrical current exclusion method (CASY counter) was used to investigate the proliferation of ReNcell VM cells. For proliferation studies ReNcell VM cells were seeded in 48-well plates, and the media was changed after 24 hours to control or EPO-containing media for 3 days and subsequently cell-counts were performed every 24 hours.

### Wst-1 assay

Metabolic activity was assessed using the reagent Wst-1 (Roche, Germany). This calorimetric assay measures the metabolic activity of viable cells based on the cleavage of the tetrazolium salt Wst-1 into formazan by mitochondrial dehydrogenases. Cells were seeded in 96-well plates and cultured under proliferating conditions and either tested during proliferation with or without hypoxia or were further used for differentiation studies. Subsequently differentiation was induced by withdrawal of the growth factors and cells were either incubated at 20% O_2 _or 3% O_2 _for an additional time period of 24 h and 72 h. The Wst-1 reagent was added to a final dilution of 1:10 for 2 h and the formazan produced by the metabolic activity of the cells was measured at a wavelength of 450 nm using a plate reader (Tecan, Mainz, Germany).

### FACS analysis

#### Cell cycle analysis

For cell cycle analysis, proliferating or differentiating cells were harvested and fixed in ice-cold 70% ethanol for 1 h at -20°C. Prior to FACS measurement fixed cells were incubated with 1 mg/ml RNase A (Sigma-Aldrich, Munich, Germany) for 30 min at 37°C following incubation with 50 μg/ml propidium iodide (PI) for 30 min at 37°C. DNA content was measured by flow cytometry (FACSCalibur, BD Bioscience, San Jose, CA, USA) and analyzed by using the Cell Quest Pro software (BD Bioscience). Aggregated cells and debris revealed by forward scattering were filtered out of the data set prior to analysis. To quantify G_1_, S, and G_2_/M populations, settings for 2N and 4N peaks were defined within each experiment from the G_1_/S-cells and applied to all samples within a given experiment.

#### Antibody staining of neuronal proteins

For the detection of βIII-tubulin positive cells, cells were detached, centrifuged at 100g at room temperature, washed with PBS without Ca^2+ ^Mg^2+ ^and fixed with 1% PFA in PBS for 15 min. Then, cells were resuspended in washing buffer (PBS + 0.5% BSA + 0.02% Na-azide) and stored at 4°C in the dark. After centrifugation cells were resuspended in saponin buffer (PBS + 0.03% Saponin + 0.5% BSA + 0.02% Na-azide) containing diluted mouse monoclonal FITC-conjugated β-III-tubulin antibody (1:100; Abcam, Cambridge, UK) and incubated for two hours at RT. Cells were washed twice with saponin buffer and resuspended in wash buffer for analysis. Measurement was done using FACSCalibur (BectonDickinson, San Jose, USA) in combination with Cell Quest Pro software.

#### TUNEL assay and staining

Apoptotic cells during differentiation were detected with an in situ cell detection kit (Roche).

Detached cells were fixed with 1% PFA/PBS for 15 min at RT. Afterwards cells were centrifuged and washing buffer was added (0.2% HSA/HBSS). Until labelling, samples were stored at 4°C. For permeabilization and labelling, samples were centrifuged and washed with PBS followed by an incubation with permeabilization solution (0.1% Triton X-100 in 0.1% sodium citrate) for two minutes on ice. After an additional washing step with PBS, cells were incubated with TUNEL reaction mixture for 1 h at 37°C at RT. As a positive control, cells were treated with DNase I for 10 minutes. As a negative control a sample treated with labelling solution was used. Subsequently cells were washed twice with PBS and a final volume of 250 μl PBS was added. The samples were measured and analysed with FACS Calibur and Cell Quest Pro Software.

### Western blot analysis

For western blot analysis total cell extracts were prepared. Cells were washed with HBSS, lysed in ice cold RIPA buffer containing protease and phosphatase inhibitor cocktails (Roche, Mannheim, Germany) and boiled 5 min at 95°C with 5× sample buffer. Protein content of lysates was determined using the bicinchoninic acid assay (BCA™, Pierce, Rockford, IL, USA). Samples were separated by SDS-PAGE with precast gels (4-15%, Bio-Rad) and subsequently the proteins were transferred to nitrocellulose membrane (Hybond-ECL, Amersham) with a semi-dry blotting system (Bio-Rad) as described [34]. Membranes were blocked with TBST containing 0.1% Tween 20 and 5% milk powder for 1 h at RT followed by incubation with primary antibodies (mouse monoclonal anti-Bcl-2 (C2), 1:500, rabbit polyclonal anti-EpoR (M20) 1:1000, mouse monoclonal anti-GAPDH 1:10,000, mouse monoclonal anti-β-actin (AC-15) 1:10,000, rabbit polyclonal anti-HIF-1α (H-206) 1:500, all Santa Cruz) overnight at 4°C in blocking buffer. Afterwards blots were rinsed 3 times with TBST (1% Tween, pH 7.6) and incubated with fluorescent dye labelled secondary antibodies (Alexa Fluor 680 goat anti-rabbit IgG, Alexa Fluor 800 goat anti-mouse IgG, Alexa Fluor 800 goat anti-rabbit IgG and Alexa Fluor 680 goat anti-mouse IgG (all 1:10,000, Molecular Probes, USA). As a molecular weight marker, the prestained peqGOLD marker IV (PEQLAB, Erlangen, Germany) was used. Visualization and quantification were performed with Odyssey Infrared Imaging System (LI-COR Biosciences GmbH, Bad Homburg, Germany). Immunoblots were scanned at a wavelength of 700 nm for Alexa Fluor 680-labeled antibodies and at a wavelength of 800 nm for IRDye 800CW-labeled antibodies, respectively using Odyssee software version 1.2. Expression of β-actin or GAPDH were used for normalization. Values were normalized and thereby relative expression levels of the target proteins were determined.

### Statistical analysis

All results are shown as mean ± SEM of data. Statistical analysis was performed with the student's t-test or One-way ANOVA with Dunnett post-test. p < 0.05 was considered to indicate statistically significance using Prism5 (GraphPad Prism. Inc., USA).

## Abbreviations

Wst: water soluble tetrazolium; NPC: neural progenitor cells; EPO: erythropoietin; EpoR: erythropoietin receptor; PFA: paraformaldehyde; PBS: phosphate buffered saline; BSA: bovine serum albumin; HSA: human serum albumin; HBSS: Hanks' balanced salt solution; RIPA: radio immuno precipitation assay; TBST: tris buffered saline with tween

## Authors' contributions

AKG: performed FACS, western blot, Wst-1 and TUNEL-assay, drafted the manuscript, participated in study design and data analysis; JF: participated in FACS experiments RH: performed western blot analysis, Wst-1 assay, read and approved manuscript; MJF, AR, SO: participated in design of study and data analysis, drafted, read and approved manuscript. All other authors participated in design of study, read and approved manuscript.
